# LTBI-negative close contacts of tuberculosis are more likely to develop the disease: enlightenment and lessons from a cluster outbreak

**DOI:** 10.3389/fpubh.2023.1136355

**Published:** 2023-07-11

**Authors:** Jingli Du, Yue Su, Enjun Dong, Juan Qiao, Ruilan Wang, Wenjuan Zhao, Jianqin Liang, Wenping Gong

**Affiliations:** Beijing Key Laboratory of New Techniques of Tuberculosis Diagnosis and Treatment, Senior Department of Tuberculosis, The Eighth Medical Center of PLA General Hospital, Beijing, China

**Keywords:** tuberculosis, close contacts investigation, cluster outbreak, LTBI, window period

## Abstract

**Background:**

Tuberculosis (TB) prevention and control among groups living together, such as students, workers, older adults in nursing homes, and prisoners, present many challenges due to their particular age and environmental factors, which can make them more susceptible to TB clusters with significant societal impact. This study aimed to evaluate a TB cluster outbreak epidemic in a university and provide suggestions for improving TB control strategies for groups living together.

**Methods:**

Pulmonary TB screening and close-contact investigation were conducted using acid-fast staining, sputum culture, GeneXpert testing, tuberculin skin testing (TST), interferon-gamma release assay (IGRA), and chest computed tomography (CT). GraphPad Prism 9.5.1 was utilized for data analysis. Collected epidemic data were comprehensively analyzed by rate comparison.

**Results:**

The TB cluster outbreak epidemic was identified with an index case confirmed positive. The initial screening was conducted on potential close contacts of the index case, and the TST’s positive rate (diameter ≥ 5 mm) and strong positive rate (diameter ≥ 15 mm) among these close contacts were 65.60% (21/32) and 34.40% (11/32), respectively. Moreover, the latent TB infection (LTBI) rate (diameter ≥ 10 mm) was 43.75% (14/32), and the IGRA’s positive rate was 9.30% (3/32). Chest CT scans did not reveal any abnormalities. Surprisingly, 5 of the close contacts developed active TB in the second screening, accompanied by changes from negative to positive TST and/or IGRA results, after 3 months of follow-up. Accordingly, we expanded the screening scope to include another 28 general contacts. We found that the positive rate (78.00%, 25/32), strong positive rate (50.00%, 16/32), and LTBI rate (62.50%, 20/32) of the 32 close contacts were significantly higher than those of the additional general contacts (28.00%, 8/28; 14.3%, 4/28; 25.00%, 7/28), as indicated by *p* < 0.05.

**Conclusion:**

In the event of an epidemic TB outbreak, it is essential to rapidly identify the source of infection and initiate timely screening of close contacts. The initial screening should be focused on individuals without LTBI, who are at higher risk of developing TB. In purified protein derivative-negative individuals living in groups, additional vaccination or revaccination with Bacille Calmette-Guérin may help prevent cluster outbreaks of TB.

## 1. Introduction

Tuberculosis (TB) is an old infectious disease caused by *Mycobacterium tuberculosis*. According to the Global Tuberculosis Report 2022 published by the World Health Organization (WHO) in October 2022, there were 10.6 million new TB cases and 0.96 TB deaths worldwide in 2021 (1). In addition, China has a large population and is among the countries with a high burden of TB, with an estimated 842,000 new cases in 2020, posing a major threat to people’s health and livelihoods (2). Furthermore, the COVID-19 pandemic has disrupted access to TB services, resulting in an increase in the incidence and mortality rates of TB, delaying global TB control by approximately 10 years (3). It is reassuring that during the COVID-19 epidemic, various countries explored many effective strategies for the prevention and control of infectious diseases, providing an example for the prevention and control of TB (4).

Bacille Calmette-Guérin (BCG) is the only vaccine for preventing TB. However, it has been reported that the protective effect of BCG vaccination only maintains for 10–15 years (5, 6), indicating that many adolescents over the age of 15 in countries with universal BCG vaccination policies may be at increased risk of *M. tuberculosis* infection. Interestingly, this age group in China tends to be gregarious university students, the new generation of migrant workers, and young freelancers. In developing countries, individuals between 25 and 44 years are prone to TB (7), which is consistent with a systematic review and meta-analysis (8).

Environmental and host factors are the two major risk factors for TB. Environmental factors determine the possibility of the first infection, whereas host factors determine the probability of post-infection morbidity. University students, the new generation of migrant workers, young freelancers, and prisoners are characterized by collective living, low income, poor living environment, heavy workload, etc. Recently, a study performed in four cities in China quantified the transmissibility of *M. tuberculosis* among students and non-students and found that TB transmissibility from the non-student to student population strongly influenced students (9). It can be concluded that these particular environmental and host conditions provide fertile ground for the outbreak and epidemic of TB in these populations (9–11).

In this study, we reported an outbreak of TB at a university in Beijing, China. We identified the individual with active TB (ATB) and conducted two rounds of screening of close contacts using etiologic, molecular, imaging, and immunologic techniques. Our results indicated that five close contacts developed ATB. This study provides a new strategy for preventing and controlling tuberculosis in cluster populations.

## 2. Materials and methods

### 2.1. Ethic declaration

The study protocol was approved by the Research Ethics Committee of the 8th Medical Center of PLA General Hospital. All procedures followed the Declaration of Helsinki and relevant policies in China. Written informed consent was obtained from all participants before enrolment.

### 2.2. Participants and groups

In May 2021, a male student with ATB was identified at a university in Beijing, China. The diagnosis of ATB was followed by the Tuberculosis Diagnostic Criteria (WS288-2017) formulated by the National Health and Family Planning Commission of China (NHFPC). To avoid the TB pandemic in students, we conducted two rounds of TB screening among close contacts. According to the degree of exposure, we divided all close contacts into two exposure levels: individuals in exposure level 1 are close contacts living in the same room with the index case or good friends of the index case, and individuals in exposure level 2 are close contacts who attended classes in the same classroom with the index case.

### 2.3. Investigation methods

**Figure 1:**
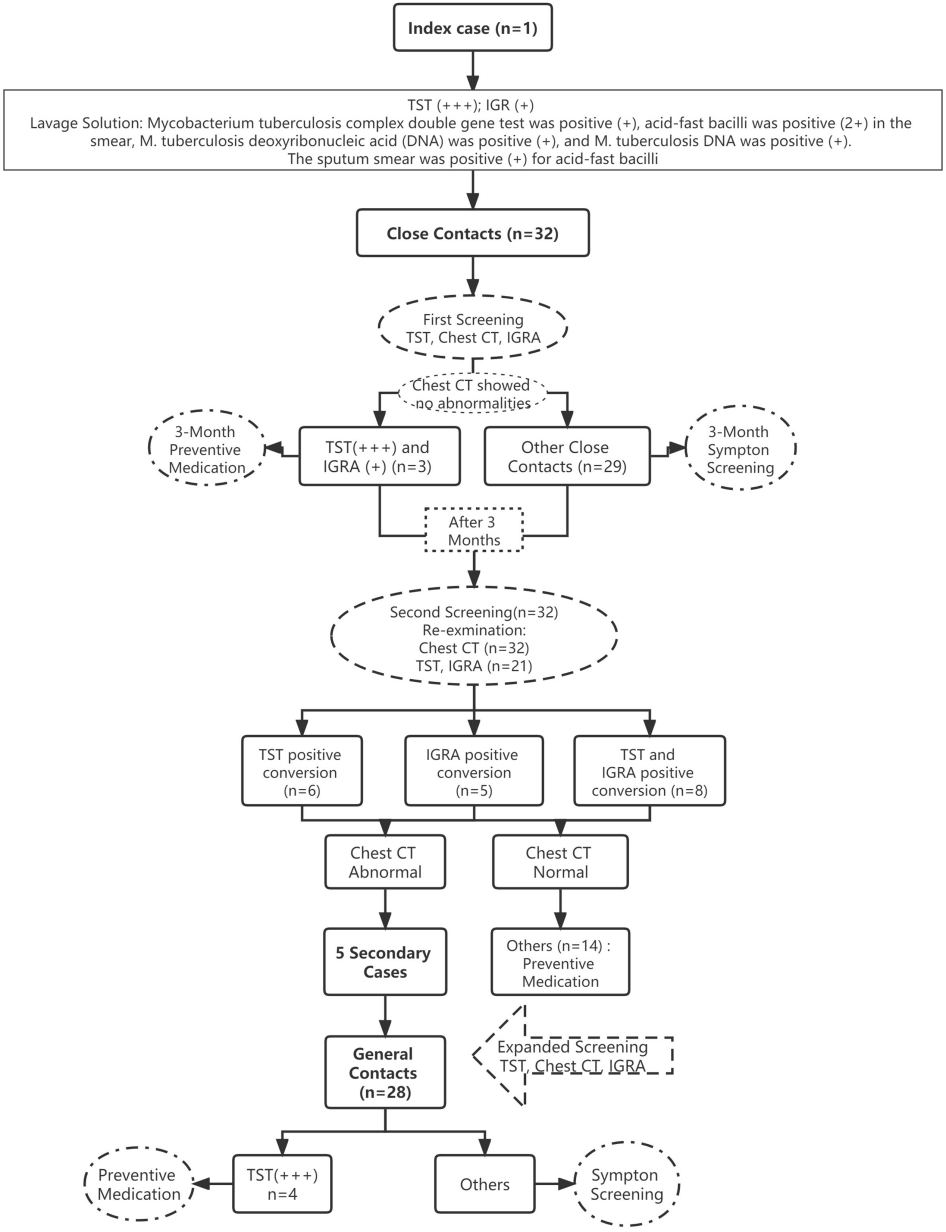
Flow chart of the disposal of this cluster outbreak of a TB epidemic.

This study examined the index case, including his basic information, onset and consultation, diagnosis and treatment, and close contacts. The close contacts were primarily screened using symptoms, *M. tuberculosis* acid-fast staining and sputum culture, GeneXpert test, chest computed tomography (CT), tuberculin skin test (TST), and interferon-γ release assay (IGRA). Preventive medication was administered to close contacts with normal chest CT, strong positive TST, and positive IGRA results. After 3 months, an additional screening was performed among close contacts with negative TST and/or negative IGRA results in the first screening. Their positive conversion rate was determined. Individuals whose TST and/or IGRA results became positive on normal chest CT were administered preventive medication. Abnormal chest radiographs and strongly positive TST results were assessed for individuals with suspicious symptoms. TB diagnosis was confirmed using smears prepared thrice, one-time culture, and molecular biological detection using the sputum or bronchoalveolar lavage fluid. Follow-up included medical observation of other screening objects for 6 months (Figure 1).

### 2.4. *Mycobacterium tuberculosis* acid-fast staining and sputum culture

Close contacts exhibiting symptoms of cough and sputum should rinse their mouths in the morning upon awakening and expectorate one or two mouthfuls of sputum forcefully. However, if the individual presents with a dry cough instead, sputum samples can be collected using the sputum induction method, where hypertonic saline is nebulized for inhalation to induce sputum production. A clean container was used for sputum collection, and approximately 0.05 mL of caseous, purulent, or otherwise suspicious sputum was selected and spread evenly onto the front of a glass microscope slide, forming an oval sputum film of 10 mm × 20 mm. The slide was allowed to air dry and heat-fixed before staining with the Ziehl-Neelsen method using an *M. tuberculosis* acid-fast staining kit (product code BA4011, BaSo, Zhuhai, Guangdong province, China), following the manufacturer’s instructions. Smear results of 1+ or more were recorded as positive for statistical analysis.

Furthermore, the rest of the sputum collected from the close contacts was utilized for sputum culture. In brief, 1–2 times the volume of 4% NaOH was added to the sputum sample, mixed using a vortex mixer, and shaken. After 15–20 min, 0.067 M phosphate buffer solution (PBS) was added and mixed. The sample was then subjected to centrifugation at 3,000 g for 20–30 min to obtain the supernatant, which was removed. The residue was mixed with 0.5 mL of PBS, and 0.1 mL to 0.3 mL of the mixture was inoculated onto a Roche culture tube (product code BA7005C-2, BaSo, Zhuhai, Guangdong province, China) and incubated at 37°C in an incubator. The tubes were observed once on the 3rd and 7th days after inoculation, and subsequently once a week until no colonies were negative at week 8, while colonies appearing positive were recorded.

### 2.5. GeneXpert test

For sputum pretreatment, one mL of sputum sample and 2 mL of 4% NaOH were added to a screw-capped pretreatment tube. The tube was tightly closed and placed in a biosafety cabinet for vortexing for 1 min to ensure complete mixing. After treatment, the samples were allowed to rest at room temperature for 15 min, followed by the addition of 45 mL of 0.067 M PBS to the liquefied sputum. The screw cap was then tightened, and the sample was centrifuged at 3,000 g for 20 min. The supernatant was discarded, and the precipitate was resuspended in 2 mL PBS. Sequentially labeled according to the specimen’s serial number, the Xpert MTB/RIF reaction box was used for testing by slowly aspirating 0.5 mL of the sputum precipitate suspension from the well of the reaction box. The lid of the box was then closed, and the GeneXpert XVI (Cepheid AB, Solna, Sweden) was used for testing, following the manufacturer’s instructions. After 2 h, the test results were read: (1) *M. tuberculosis* Ct value less than 16 was considered high; (2) Ct value 16–22 was considered intermediate; (3) Ct value 22–28 was considered low; (4) Ct value greater than 28 was considered very low. A sample with a Ct value less than 28 was reported as *M. tuberculosis* positive, and a Ct value higher than 28 was declared as *M. tuberculosis* negative.

### 2.6. IGRA

A total of 5 mL of venous blood was collected from each participant using heparin-anticoagulated vacuum collection tubes, gently inverted to mix the anticoagulant and blood. Three separate culture tubes were prepared: T-tube containing *M. tuberculosis*-specific antigens ESAT-6 and CFP-10, N-tube as a background control without any antigens, and P-tube as a positive control supplemented with the non-specific stimulating antigen plant hemagglutinin (PHA). One mL of the blood sample prepared above was added to each tube, gently mixed, and incubated for 22 h in an incubator at 37°C. The supernatant was collected after centrifugation at 1,000 g for 10 min. The IFN-γ concentration was detected by a TB-IGRA Detection Kit (Beijing Wantai Biological Pharmaceutical Co., LTD, Beijing, China) following the manufacturer’s instructions. The results were scored using a microplate reader with a single filter (450 nm) or a dual filter (450 nm, 600–650 nm). According to the manufacturer’s instructions, the TB-IGRA assay result was deemed positive if the value of T-N was ≥14 and N/14 (T: concentration of testing tube; N: concentration of background control tube) (12, 13).

### 2.7. Definitions and statistical analysis

#### 2.7.1. Index case

The index case is an initially identified case of new or recurrent TB in a person of any age in a specific household or another comparable setting in which others may have been exposed (14).

#### 2.7.2. Cluster epidemic situation

For a highly concentrated unit, such as schools, two or more cases of TB with epidemiological correlation, which occurred within 3 months, resulted in a TB cluster epidemic.

#### 2.7.3. Secondary confirmed cases

Secondary confirmed cases met the Pulmonary TB Diagnosis Criteria WS288-2017 and were epidemiologically related to the index case during the cluster epidemic period (May to December 2021).

#### 2.7.4. Standard of the criterion for the TST result

Tuberculin skin test method: Briefly, 5 IU tuberculin purified protein derivative (PPD) was injected into the anterior one-third center of the palmar side of the left forearm, and skin induration was examined after 72 h according to the Technical Guidelines for tuberculosis Prevention and Control in China 2021 (15). The TST result is interpreted as follows: (1) Negative (−), the mean induration diameter is less than 5 mm, or no reaction was observed; (2) Generally positive (+), the mean induration diameter is ≥5 mm but <10 mm; (3) Moderately positive (++), the mean induration diameter is ≥10 but <15 mm; (4) Strongly positive (+++), the mean induration diameter is ≥15 mm or the skin at the injection site has local double circles, bullae, necrosis, and lymphangitis. The judgment principle for LITBI: no BCG vaccination and interference from non-tuberculosis mycobacteria (NTM), TST ≥ 5 mm; And BCG vaccinators and/or NTM endemic areas, TST ≥ 10 mm. Definition of positive TST conversion: If the previous TST result was less than 5 mm, the repeat test result should be ≥10 mm; if the previous test had a TST result of 5–10 mm, the repeat test result should increase by 10 mm or more.

#### 2.7.5. Criteria for close contacts

The criteria for judgment and screening of close contacts are based on “the expert consensus on epidemiological investigation and on-site management of school tuberculosis outbreaks” (16). Index cases exhibiting positive etiology, pulmonary cavity, or symptoms of pulmonary TB should adhere to the following criteria: personnel who were in direct contact with the index case for 8 h or more, or whose cumulative contact time reached or exceeded 40 h, in the same class, dormitory, or other enclosed spaces within the 3 months preceding diagnosis until 14 days post-treatment. Conversely, an index case with negative etiology, no cavity, and no symptoms should meet the following criteria: personnel in the same class and dormitory as the index case from 1 month before diagnosis until 14 days post-treatment. In the event that new TB cases are identified among close contacts, the screening parameters of contacts will be expanded according to the level of exposure. Individuals working in the same office building and/or residing on the same dormitory floor would be considered general contacts.

#### 2.7.6. Statistical analysis

Data in this study were analyzed using GraphPad Prism software 9.5.1 version (San Diego, CA, United States). Fisher’s exact test was used for categorical variables with small sample sizes, and the chi-square test was used for categorical variables with large sample sizes. A *p-*value of less than 0.05 was considered a significant difference. Furthermore, the calculations of the relative risks (RR) with a 95% confidence interval (CI) were done by Poisson regression. Exact Poisson regression was used when a cero frequency was found. The program Stata^®^ (Stata Corp., College Station, Texas, United States) was used for this analysis (17).

## 3. Results

### 3.1. Information of the index case

**Figure 2:**
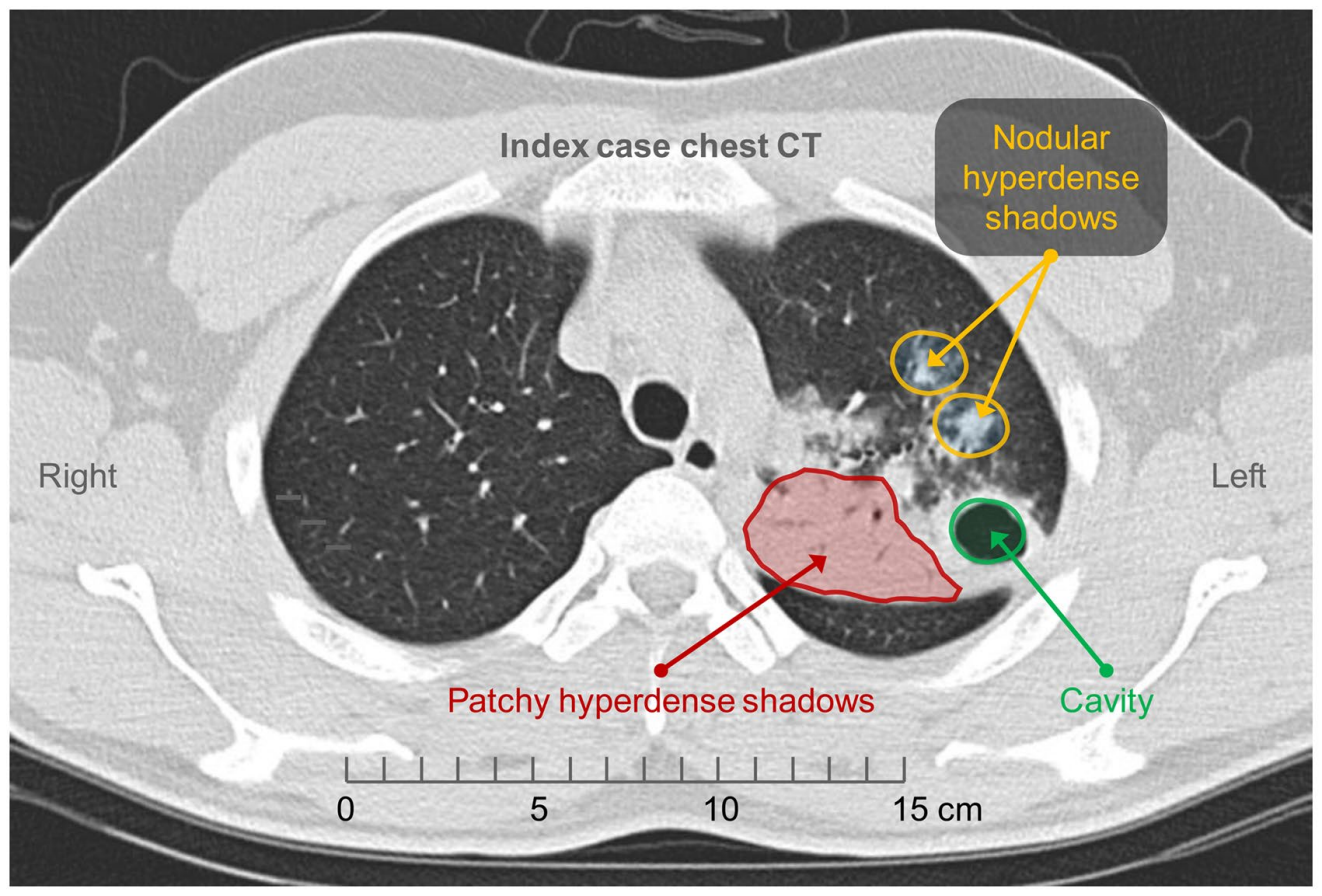
Chest CT of the index case. Patchy and nodular hyperdense shadows with cavity formation in the upper lobe of the left lung.

The index case was a 23-year-old male patient that developed chest pain and intermittent fever in May 2021 without prominent cough, expectoration, or other symptoms. Chest CT revealed patches and nodular hyperdense shadows with cavity formation in the upper lobe of the left lung (Figure 2). The following results were obtained from the examination of the blood sample: (1) C-reactive protein, 26.30 mg/L ↑; (2) Blood routine examination: neutrophil percentage, 73.00% ↑, lymphocyte percentage, 16.50% ↓; (3) Erythrocyte sedimentation rate (ESR), 31 mm/h ↑; (4) procalcitonin, <0.05 ng/mL; and (5) carbohydrate antigen, 125 50.67 U/mL ↑. No obvious abnormalities were found in the antineutrophil plasma antibody, autoantibody spectrum, or blood culture.

The patient underwent a tracheoscopy on May 19, 2021. However, no significant abnormalities were found. The following results were obtained using the lavage solution: *Mycobacterium tuberculosis* complex double gene test, positive (+); acid-fast bacilli, positive (2+) in the smear; *M. tuberculosis* deoxyribonucleic acid (DNA), positive (+); rifampicin *rpoB*, not mutated; non-*M. tuberculosis* DNA, negative (−); and *M. tuberculosis* DNA, positive (+). The sputum smear was positive (+) for acid-fast bacilli.

### 3.2. The results of symptoms, TST, IGRA, and chest CT in close contacts screened in the first round of screening

The index case was a patient with a positive TB etiology and lung cavities. According to the criteria for a close-contact population, 32 screening objects were primarily identified. The detailed information of these 32 close contacts can be found in Table 1. Furthermore, we also determined the symptom, chest CT, TST, and IGRA of these close contacts. Our results showed that the positivity rate of symptom screening was 0.00% (0/32), the positivity rate of TST (+, ++, and +++) was 65.60% (21/32), the strong positivity rate of TST (+++) was 34.40% (11/32), and the latent infection rate of TST (++ and +++) screening was 43.75% (14/32) (Table 2). Furthermore, we also found that the positivity rate of IGRA and chest CT in 32 close contacts were 9.30% (3/32) and 0.00% (0/32), respectively (Table 2). Based on these screening results, three close contacts with a strong positivity rate of TST (+++) and a positivity rate of IGRA received prophylactic medication, while the remaining close contacts were followed up for 3 months.

**Table 1:** General information of 32 close contacts of the index active TB case.

Case No.	Age (years)	Gender	Residence area	Exposure level^a^	Suspicious symptoms of TB	BCG scar	Size of PPD (mm × mm)	Judgment of PPD result	Latent of TB	IGRA	Chest CT	Preventive therapy^c^
1	33	Male	Rural	2	No	Yes	17 × 17	**+++**	+	+	−	Yes
2^b^	24	Male	Rural	1	No	Yes	5 × 5	**+**	−	−	−	No
3	21	Male	Urban	2	No	Yes	5 × 6	**+**	−	−	−	No
4	26	Male	Rural	2	No	Yes	5 × 5	**+**	−	−	−	No
5	26	Male	Rural	2	No	No	12 × 13	**++**	+	−	−	No
6^b^	24	Male	Urban	1	No	Yes	5 × 5	**+**	−	−	−	No
7	20	Male	Urban	1	No	No	15 × 16	**+++**	+	+	−	Yes
8	23	Male	Rural	2	No	Yes	0 × 0	**−**	−	−	−	No
9	20	Male	Rural	1	No	Yes	0 × 0	**−**	−	−	−	No
10	28	Male	Urban	2	No	No	0 × 0	**−**	−	−	−	No
11	24	Male	Urban	2	No	Yes	0 × 0	**−**	−	−	−	No
12^b^	21	Male	Urban	1	No	No	0 × 0	**−**	−	−	−	No
13^b^	21	Male	Rural	1	No	No	0 × 0	**−**	−	−	−	No
14	26	Male	Urban	2	No	Yes	11 × 12	**++**	+	−	−	No
15^b^	23	Male	Rural	1	No	Yes	0 × 0	**−**	−	−	−	No
16	23	Male	Rural	2	No	Yes	13 × 14	**++**	+	−	−	No
17	20	Male	Urban	2	No	Yes	8 × 8	**+**	−	−	−	No
18	26	Male	Rural	2	No	No	0 × 0	**−**	−	−	−	No
19	28	Male	Urban	2	No	No	0 × 0	**−**	−	−	−	No
20	35	Male	Urban	2	No	Yes	0 × 0	**−**	−	−	−	No
21	29	Male	Urban	2	No	Yes	5 × 5	**+**	−	−	−	No
22	25	Male	Rural	2	No	Yes	7 × 7	**+**	−	+	−	No
23	35	Male	Urban	2	No	Yes	0 × 0	**_**	−	−	−	No
24	21	Male	Rural	2	No	Yes	18 × 20	**+++**	+	−	−	Refuse
25	33	Male	Rural	2	No	Yes	17 × 18	**+++**	+	−	−	Refuse
26	21	Male	Rural	2	No	Yes	17 × 17	**+++**	+	−	−	Refuse
27	31	Male	Urban	2	No	Yes	15 × 16	**+++**	+	−	−	Refuse
28	35	Male	Urban	2	No	Yes	17 × 17	**+++**	+	−	−	Refuse
29	33	Male	Urban	2	No	Yes	15 × 15	**+++**	+	−	−	Refuse
30	31	Male	Urban	2	No	Yes	20 × 20	**+++**	+	−	−	Refuse
31	30	Male	Urban	2	No	Yes	19 × 19	**+++**	+	−	−	Yes
32	35	Male	Rural	2	No	Yes	15 × 15	**+++**	+	−	−	Refuse

a Individuals in exposure level 1 are close contacts living in the same room with the index case or good friends of the index case, and individuals in exposure level 2 are close contacts who attended classes in the same classroom with the index case.

b Close contacts developed active TB after 3 months.

c Yes, the current evidence supports preventive anti-tuberculosis treatment for close contact and the close contact agreed and received preventive therapy; Refuse, the current evidence supports preventive anti-tuberculosis treatment for close contacts but the close contact did not consent to prophylactic treatment; No, the current evidence does not support preventive anti-tuberculosis treatment for close contact and the close contacts did not receive preventive therapy.

**Table 2:** First-round screening of the close contacts of the index case.

Item	TST				IGRA		Chest CT	
	−	+	++	+++	−	+	−	+
Close contacts	34.40% (11/32)	21.80% (7/32)	9.40% (3/32)	34.40% (11/32)	90.60% (29/32)	9.40% (3/32)	100% (32/32)	0.00% (0/32)

**Figure 3:**
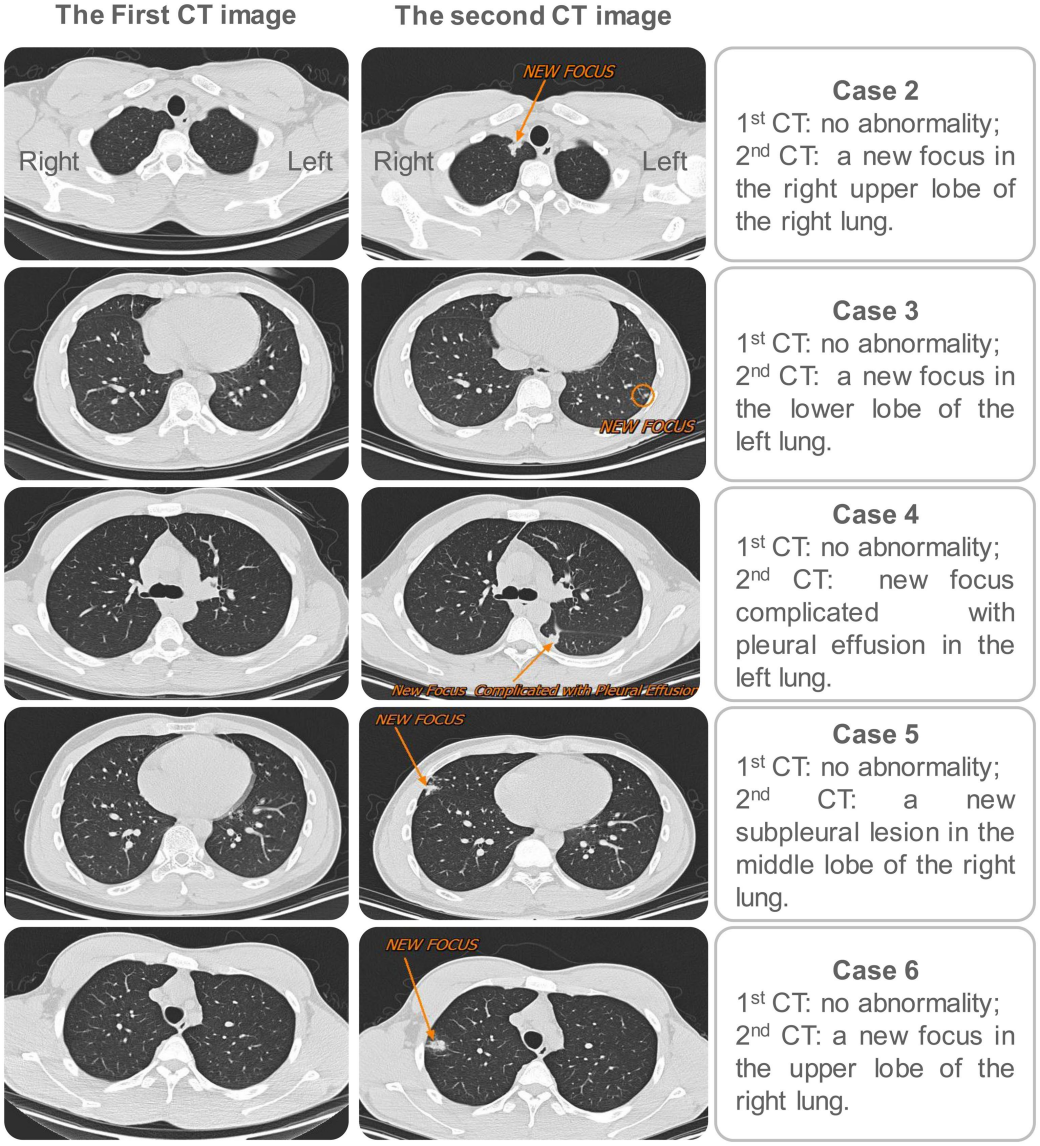
Comparison of the first and second screenings of chest CT for the five secondary cases. In the first screening, no abnormalities were found in the chest CT for the 5 cases. Three months later, the second screening of chest CT was performed, and new lesions were observed in the lungs of all five cases. Left: the first CT image for each case; Right: the second CT image for each case.

### 3.3. The results of TST, IGRA, and chest CT in general contacts and close contacts screened in the second round of screening

In the second round, we expanded screening from close contacts who were screened in the first round to general contacts. Our results showed that (Table 3): (1) negative (−), generally positive (+), moderately positive (++), and the strongly positive (+++) rate of TST in close contacts were 21·90% (7/32), 15.60% (5/32), 12.50% (4/32), and 50.00% (16/32), and these in general contacts were 71.40% (20/28), 3.60% (1/28), 10.70% (3/28), and 14.30% (4/28); (2) positive rate of IGRA and Chest CT in close contacts were 25.00% (8/32) and 15.70% (5/32), and these in general contacts were 10.70% (3/28) and 0.00% (0). These data suggest that close contacts of the index case have higher positive rates of TST, IGRA, and chest CT compared with the general contacts of the index case.

**Table 3:** TST, IGRA, and chest CT results of the second-round screening of the close and general contacts of the index case.

Item	TST				IGRA		Chest CT	
	−	+	++	+++	−	+	−	+
Close contacts of the index case	21·90% (7/32)	15.60% (5/32)	12.50% (4/32)	50.00% (16/32)	75.00% (24/32)	25.00% (8/32)	84.40% (27/32)	15.70% (5/32)
General contacts of the index case	71.40% (20/28)	3.60% (1/28)	10.70% (3/28)	14.30% (4/28)	78.10% (25/28)	10.70% (3/28)	100.00% (28/28)	0.00% (0)

### 3.4. Comparison of TST, IGRA, and chest CT results of general contacts and close contacts screened in the second round of screening

We further compared the results of TST, IGRA and chest CT between general contacts and close contacts screened in the second round of screening. Our results showed that (Table 4): (1) The risk of TST positive (diameter ≥ 5 mm) in close contacts of the index case was 2.73 times higher than that in general contacts (*p* = 0.013, RR 2.73, 95%CI: 1.23–6.06); (2) The risk of TST strong positive (diameter ≥ 15 mm) in close contacts of the index case was 3.5 times higher than that in general contacts (*p* = 0.025, RR 3.50, 95%CI: 1.17–10.47); (3) The risk of latent TB infection (diameter ≥ 10 mm) in close contacts of the index case was 2.5 times higher than that in general contacts (*p* = 0.037, RR 2.50, 95%CI: 1.06–5.91). In addition, we found that the risk of IGRA positivity or abnormal chest CT in close contacts of the index patient was not significantly different from that in general contacts (Table 4).

**Table 4:** Comparison of screening results between close and general contacts of the index case.

Factor	Subgroup	Close contacts of the index case (%)	General contacts of the index case (%)	Relative risk	95% CI	*p*-value
TST positive rate	≥5 mm	25 (78.12%)	8 (28.57%)	2.73	1.23–6.06	**0.013**
	<5 mm	7 (21.88%)	20 (71.43%)			
TST strong positive rate	≥15 mm	16 (50.00%)	4 (14.29%)	3.50	1.17–10.47	**0.025**
	<15 mm	16 (50.00%)	24 (85.71%)			
TST latent infection rate	≥10 mm	20 (62.50%)	7 (25.00%)	2.50	1.06–5.91	**0.037**
	<10 mm	12 (37.50%)	21 (75.00%)			
IGRA	+	8 (25.00%)	3 (10.71%)	2.33	0.62–8.80	0.211
	−	24 (75.00%)	25 (89.29%)			
Chest CT	Abnormal	5 (15.63%)	0 (0.00%)	5.88	0.80 to +infinity	0.086
	Normal	27 (84.37%)	28 (100.00%)			

TST, tuberculin skin testing; IGRA, interferon-gamma release assay. *p* < 0.05.

### 3.5. Secondary cases

**Figure 4:**
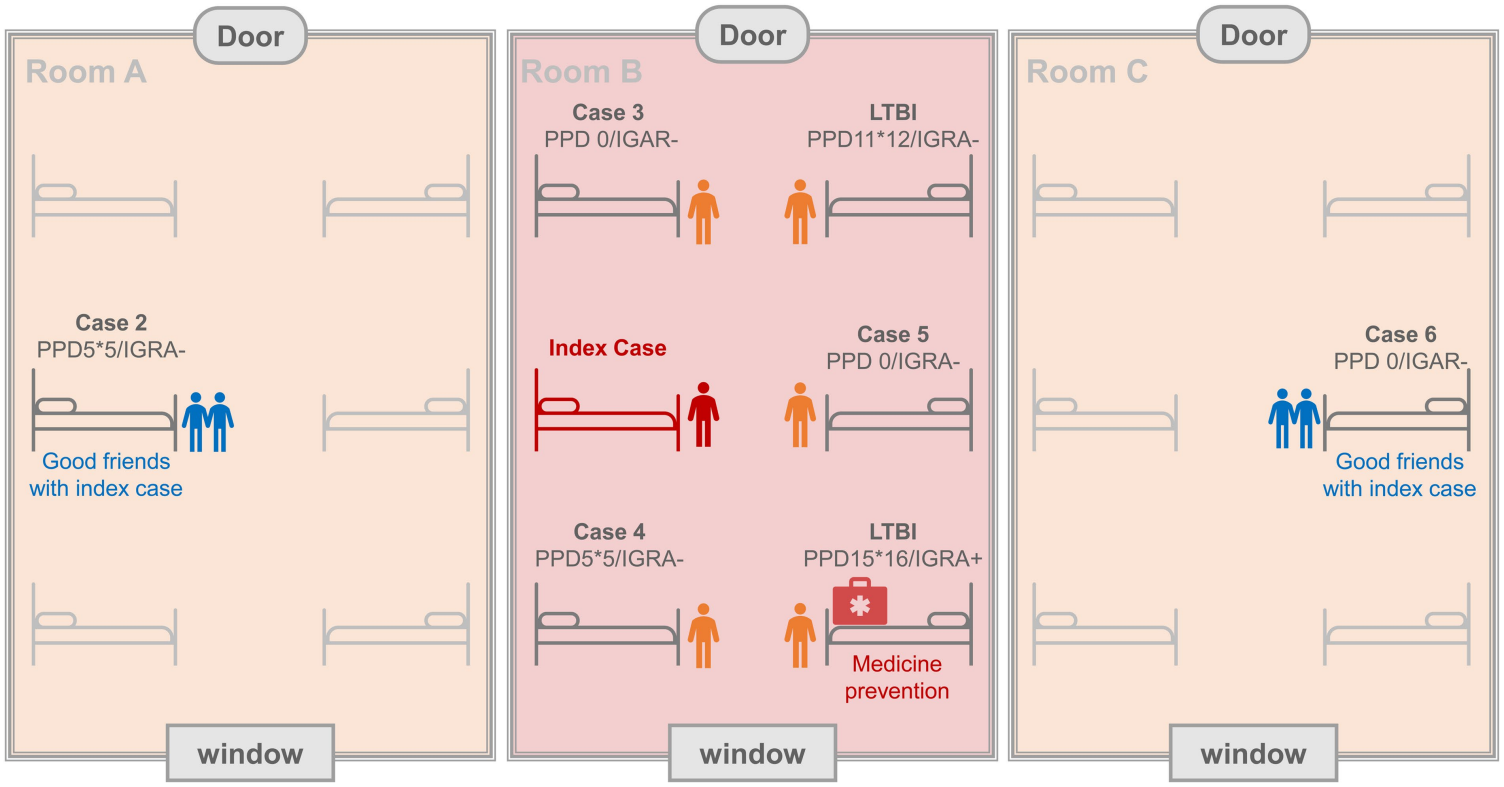
Schematic representation of the living environment of five secondary cases and index cases. In the first round of screening, we identified two individuals with LTBI who shared a room with the index patient, one of whom received prophylaxis because of a strongly positive PPD and IGRA(+). In the second round of screening, we identified 5 close contacts who developed ATB (secondary cases), including case 2, case 3, case 4, case 5, and case 6. Interestingly, case 3, case 4, and case 5 lived in the same dormitory (Room B) with the index case, and case 2 (Room A) and case 6 (Room C) were close friends who did not live in the same dormitory with the index case.

Five secondary cases (case 2, case 3, case 4, case 5, and case 6) were found in the second round of screening (Figure 3). By comparing the first and second screening results of the 5 secondary cases, we found that the TST results of all 5 secondary cases changed from negative or weakly positive to strongly positive, the IGRA results suggested that case 4, case 5, and case 6 changed from negative to positive, and the chest CT indicated that all 5 secondary cases had lung injury (Table 5). In addition, the pathogenic diagnostic results revealed positive sputum LAMP result in case 3, positive sputum culture and Xpert results in case 4, and positive BALF LAMP result in case 5 (Table 5). Furthermore, we also conducted an epidemiological investigation. During the 3-month symptom monitoring, there were no obvious symptoms such as cough, expectoration, and fever in these 5 secondary cases. Only case 4 experienced chest pain and fever at the 11th week. Of these 5 secondary cases, 3 were in the same dormitory with index case, while the other two cases from different dormitories were also good friends of index case and often stayed together (Figure 4). These results suggest that the risk of exposure was highest among persons living in the same room with index case, but that the LTBI population may have some resistance to transmission from the index case. In addition, we found that even people who did not live in the same room were susceptible to infection if they had frequent, unprotected interactions with index case.

**Table 5:** Detailed information of the five secondary cases.

Case No.	TST (diameter × transverse diameter mm)		IGRA (pg/mL)		Chest CT		Pathogen diagnosis
	May 27	Sep 17	May 27	Sep 17	May 27	Sep 17	
Case 2	0	20 × 21	4	2	–	New lesions in the upper lobe of the right lung	–
Case 3	5 × 5	20 × 20	1	1	–	New lesions in the outer basal segment of the lower lobe of the left lung	Sputum LAMP (+)
Case 4	5 × 5	15 × 16	0	20	–	A new lesion in the dorsal segment of the lower lobe of the left lung with a little pleural effusion	Sputum culture (+) XPERT (+)
Case 5	0	16 × 16	0	47	–	New subpleural lesion in the middle lobe of the right lung	BALF LAMP (+)
Case 6	0	17 × 18	6	322	–	New lesion in the upper lobe of the right lung	–

### 3.6. Analysis of TB risk factors of secondary cases

To further determine the potential risk factors for active TB in close contacts of the index case, we analyzed the factors (age, BCG scar, residence area, and exposure levels) in 32 close contacts with or without LTBI and active TB. Our results showed that among these four factors, the exposure level was the significantly higher risk factor for the occurrence of secondary cases (*p* = 0.030, RR = 13.5, 95% CI: 1.21–141.77, Table 6).

**Table 6:** Risk factors for active TB in close contacts of the index case.

Terms	Latent TB		*p*-value^b^	Active TB		*p*-value
	−	+		−	+	
**Age**						
20–29	16	6	0.0494	17	5	0.1550^b^
30–38	3	7		10	0	
**BCG scar**						
Yes	13	12	0.4264	22	3	0.2964^b^
No	5	2		5	2	
**Residence area**						
Urban	17	10	1.0000	15	2	0.6454^b^
Rural	15	8		12	3	
**Exposure level**^a^						
1	6	1	0.1037	2	5	0.0030^c^
2	12	13		25	0	RR = 13.5 (95% CI 1.21–141.77)^c^

a Exposure level 1 represents a close contact living in the same room as the ATB case or a good friend of the index case, exposure level 2 represents close contacts who attended classes in the same classroom as the index case.

b These data were analyzed with the Fisher’s exact test using GraphPad Prism 9.5.1 Version (San Diego, CA, United States).

c The RR of the exposure level was estimated by Exact Poisson regression using Stata^®^ (Stata Corp., College Station, Texas, United States).

### 3.7. Lessons and recommendations

**Figure 5:**
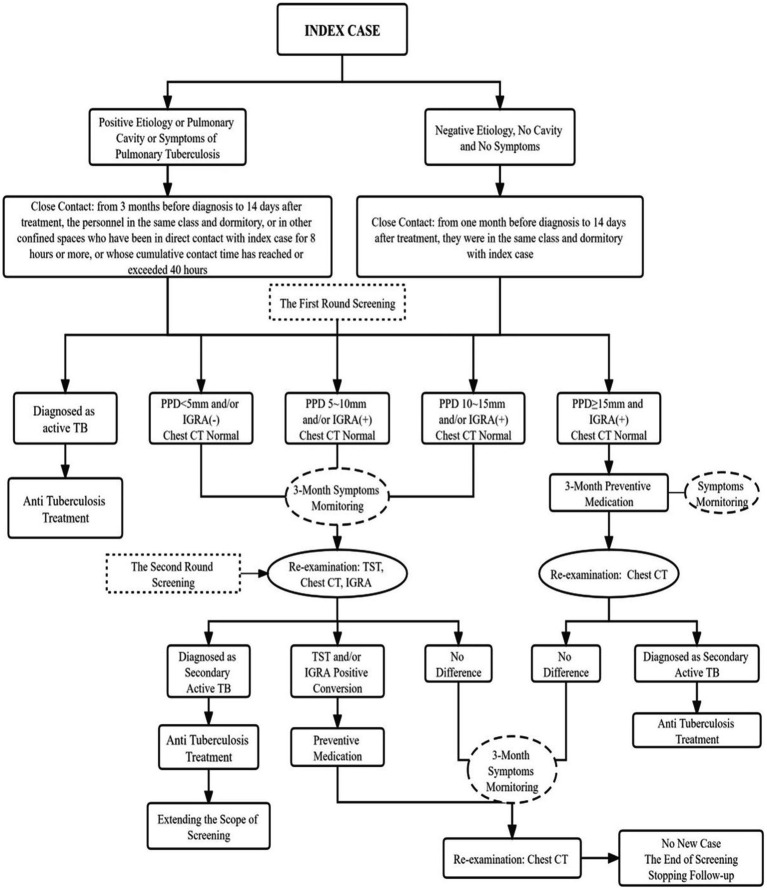
Epidemiologic investigation of the tuberculosis outbreak and the process of screening, diagnosis, prevention and treatment of index cases and close contacts.

According to the handling of the above TB epidemic situation, the scope of close contacts and the appropriate handling process should be determined according to whether the indicated case is infectious. The specific process is shown in Figure 5.

## 4. Discussion

We reported an outbreak of tuberculosis in Beijing, China. Since the index case sought medical treatment due to symptoms in May 2021, an epidemiological investigation was quickly carried out. The index case was a university student with cavitary pulmonary TB diagnosed by the positive sputum bacteria and strong infectivity. To minimize transmission from the index patient to close contacts, we conducted a thorough search of all contacts of the index patient from 3 months before diagnosis to 14 days after treatment initiation and identified 32 close contacts. In this study, the close contacts should be individuals shared the same class or dormitory with the index case, have been direct contacted with the index case in other confined spaces for more than 8 h, or have been in contact with the index case more than 40 h. TST, chest CT, and IGRA were performed for the close contacts. The latent infection rate of the TST screening (above moderately positive) was 43.75% (14/32) and the IGRA positivity rate was 9.30% (3/32). No abnormalities were found via chest CT screening. According to the technical guidelines for tuberculosis prevention and control, TST positive and IGRA positive contacts gave isoniazid (0.3 g once daily) and rifampicin (0.6 g once daily) preventive treatment for 3 months (15). The second round of screening was performed 3 months after symptom monitoring. In some countries, the second screening is made 8–10 weeks after the first (18). Five secondary cases were found, all of which were previously IGRA negative and/ or TST negative (two cases with TST 5 mm × 5 mm had a history of BCG injection in childhood) and were TST strongly positive and IGRA positive in the second round of screening. Accordingly, close contacts were identified to be in the window period for TB infection at the time of the first screening. Thus, the window period for TB infection requires more vigilance to manage TB epidemic in the army, school, or other collective living groups. In addition, the population without LTBI in the initial screening stage should receive more attention than the individuals with LTBI, as their risk of TB is more significant.

In the fourth national epidemiological survey conducted in China in 2000, the estimated natural infection rate of TB in the entire age group was 44.50% based on TST. In 2013, Gao et al. performed a population-based, multicenter, prospective study on LTBI. Based on their findings, TST might overestimate the LTBI rate in the Chinese population compared with IGRA. The TST positivity rate (TST ≥ 10 mm) ranged from 15.50 to 41.70% after age and sex standardization, and the IGRA positivity rate ranged from 13.50 to 19.80% (19). The IGRA positivity rate of the close-contact population of patients with TB was relatively higher (32.00–48.00%) (13, 20). Wu et al. (21) reported that the TST and IGRA positivity rates of newly recruited soldiers in the Chinese army were 41.00 and 21.00%, respectively. When the latent *M. tuberculosis* infection rate of close contacts screened in this study was lower than or equal to that of the general population, the screening period of some TST-negative close contacts may have coincided with the window period of LTBI. Therefore, interventions comprising 3 months of symptom monitoring, 3 months of re-examination after the initial screening, and preventive treatment only for close contacts with strong TST positivity would ignore some hidden secondary cases and affect the prevention and control effectiveness of the epidemic. Currently, the scope of such screenings is limited. As a result, when the close contacts were screened for collective living groups with etiologically positive and highly infective indicator cases, and their screening period was in the window period, it was unclear whether the scope of preventive medication should be reconsidered according to the degree of their close contact with the index cases. The five patients newly diagnosed after our second screening were all Level 1 close contacts (roommates, close friends) of the index case. Hence, as the screening period was in the window period of tuberculous infection, preventive medication should be administered according to the degree of close contact, regardless of the basis for tuberculous infection, at least for the same dormitory personnel. Three months after the first screening, the second screening found five cases of TB with pulmonary lesions, one with pleural effusion. During this period, we conducted symptom monitoring, there were no obvious symptoms such as cough, sputum and fever in these 5 secondary cases. Only case 4 had chest pain and fever in week 11. In some countries, the second screening is performed 8–10 weeks after the first (18). Generally, the close contacts would receive chest CT once every 3 months to explore new cases, considering the window period of LTBI and it would have high risk of TB infection during this time, 3 months was too long to control TB transmission of new cases. Therefore, the close contacts should be subjected to chest x-ray or CT once a month to detect new cases, because chest x-ray would not show some initial and small lesions in the lung, leading to missed diagnosis, and CT may cause high-dose radiation exposure. Low-dose spiral CT would be recommended for monthly re-screening to detect and treat the disease earlier, effectively block the epidemic and control the spread of the disease.

Bacille Calmette-Guerin (BCG) is the only approved vaccine used to prevent TB, its target population includes infants and newborns. However, the preventive effect of BCG vaccine against TB is limited. Continuous stimulation can only induce short-lived effector memory T cells and effector T cells (22). Therefore, the immune effect of BCG weakens with age increasing. In 1995, the World Health Organization highlighted the lack of a scientific basis for BCG revaccination, and the Chinese Health Commission was discontinued in 1997. Nevertheless, some countries still selectively vaccinate high-risk populations, including close contacts with TB. Further, the immune effect of revaccination has been proven to be effective. BCG vaccines can induce immunity training to enable nonspecific immune protection, and this protective effect is continuous (23, 24). According to some studies, revaccination with BCG could reduce the likelihood of TB transmission, with the risk of infection reduced from 5.70% per year to 4.80% per year; infection could be avoided in 17.00% of cases (25). We have analyzed the relationship between a history of BCG vaccination and LTBI and found that there was no significant relationship between a history of BCG vaccination and latent infection, as the protective effect of BCG generally lasts for 5–10 years. Although previous studies have shown that young women living in rural areas, those living in western China, and those without a history of BCG vaccination are more likely to develop severe TB (26), the protective effect of BCG in adults is poor (27, 28). Hence, all collective living groups (students, workers, older adults living in nursing homes, and prisoners) should receive TB screening, and personnel without a latent infection should be revaccinated with BCG. Such intervention may reduce the likelihood of a TB outbreak.

During the management of this epidemic, we expanded the scope to screen 28 general contacts after new cases were identified during the second screening. Among the 28 patients, the positivity rate of TST was 28.60% (8/28), strong positivity rate of TST was 14.30% (4/28), and latent infection rate of TST screening (above moderate positivity) was 25.00% (7/28). The IGRA positivity rate was 10.70% (3/28). The positivity rate of CT screening was 0.00% (0/28). The TST positivity rate (78.00%), latent infection rate (62.50%), and strong positivity rate (50.00%) of close contacts were significantly higher than those of general contacts (*p* < 0.05). The positivity rate of IGRA in close contacts was 25.00% and higher than that in general contacts (10.70%), however, the difference was not statistically significant, which might be related to the small sample size. IGRA is less sensitive than TST in terms of its screening effects on the latent infection of tuberculous bacteria; however, its specificity is high (29). The detection of TST is affected by many factors, such as BCG vaccination, environmental non-TB mycobacteria (NTM), and host immune status. The specificity of the results was poor and false positives were detected (30). Although IGRA was not affected by BCG and most NTM (12, 31), its sensitivity was low. The five secondary cases of active pulmonary TB showed a strongly positive reaction based on TST detection, and two remains IGRA negative, which is inconsistent with the results of previous studies. We considered that IGRA was detected using an *in vitro* experiment, which might have different degrees of false-negative reactions owing to the influence of many factors, such as blood sample collection, blood sample processing, experimental platforms, artificial technical operations, and reagents. Therefore, the combined application of the TST and IGRA in the screening of a close-contact population of TB can improve the sensitivity and specificity of screening for latent TB infection and can achieve complementary advantages to a certain extent.

This study also has some limitations: (1) How the index case became infected with *M. tuberculosis* was not clear; (2) Although this study analyzed the risk of age, BCG scarring, area of residence, and exposure level for close contacts of reported cases, it did not evaluate the role of risk factors such as body mass index, smoking, alcohol consumption, use of other drugs, and disease in the development of secondary cases in close contacts; (3) The differences in plasma cytokine levels and the absolute number and percentage of lymphocyte subsets in the peripheral blood of close contacts in the first and second screening rounds were not analyzed, thus missing the opportunity to further analyze the etiology of secondary cases; (4) The conclusions and recommendations of this study are based on a study with a small sample size, and studies with larger sample sizes are needed to verify our conclusions.

## 5. Conclusion

In summary, we addressed a TB cluster outbreak and conducted two rounds of screening for the close contacts of a diagnosed TB case. The results evidently differed from those of previous studies. After the second screening, five secondary cases of pulmonary TB were found to have a negative result (TST/IGRA/chest CT) in the first screening. Thus, the window period of LTBI in close-contact screening should be prioritized, and more attention should be paid to people without any LTBI in the preliminary screening, whose risk of pulmonary TB would be more significant owing to a lack of TB antibody. Accordingly, it is worth considering whether the interval between the two screenings should be shortened and whether the indication of preventive medication should be changed. Thus, in addition to individuals who are strongly positive, other individuals should also receive preventive medication according to their degree of exposure.

## Data availability statement

The original contributions presented in the study are included in the article/supplementary material, further inquiries can be directed to the corresponding authors.

## Ethics statement

The studies involving human participants were reviewed and approved by Research Ethics Committee of the 8th Medical Center of PLA General Hospital. The patients/participants provided their written informed consent to participate in this study. Written informed consent was obtained from the individual(s) for the publication of any potentially identifiable images or data included in this article.

## Author contributions

JD, WG, and JL: conceptualization. JD, YS, ED, RW, WZ, and JQ: methodology. JD, YS, and WG: data analysis. JD and YS: writing of the original manuscript. WG and JL: review and revision. All authors contributed to the article and approved the submitted version.

## Funding

This work was supported by the Special Research on Health and Epidemic Prevention (Grant No. 22FYFH13).

## Conflict of interest

The authors declare that the research was conducted in the absence of any commercial or financial relationships that could be construed as a potential conflict of interest.

## Publisher’s note

All claims expressed in this article are solely those of the authors and do not necessarily represent those of their affiliated organizations, or those of the publisher, the editors and the reviewers. Any product that may be evaluated in this article, or claim that may be made by its manufacturer, is not guaranteed or endorsed by the publisher.

## Abbreviations

BCG: Bacille Calmette-Guerin
CA125: Carbohydrate antigen 125
CRP: C-reactive protein
IGRA: Interferon-gamma release assay
LTBI: latent TB infection
NTM: Non-tuberculous mycobacteria
PCT: Procalcitonin
PPD: Purified protein derivative
TB: Tuberculosis
TST: Tuberculosis skin test
WHO: World Health Organization

## References

[1] WHO. Global tuberculosis report 2022. World Health Organization: Geneva, (2022).

[2] WHO. Global tuberculosis report 2021. Geneva: World Health Organization (2021).

[3] Dheda K Perumal T Moultrie H Perumal R Esmail A Scott AJ. The intersecting pandemics of tuberculosis and COVID-19: population-level and patient-level impact, clinical presentation, and corrective interventions. Lancet Respir Med. (2022) 10:603–22. doi: 10.1016/S2213-2600(22)00092-3 35338841 PMC8942481

[4] Dheda K Pinto L Mutsvangwa J Leung CC von Delft A Ruhwald M. Accelerate investment and action to find the missing patients with tuberculosis. Lancet. (2022) 399:2086–8. doi: 10.1016/S0140-6736(22)00535-9 35334210 PMC8940183

[5] Gong W Pan C Cheng P Wang J Zhao G Wu X. Peptide-based vaccines for tuberculosis. Front Immunol. (2022) 13:830497. doi: 10.3389/fimmu.2022.830497 35173740 PMC8841753

[6] Gong W Liang Y Wu X. The current status, challenges, and future developments of new tuberculosis vaccines. Hum Vaccin Immunother. (1697-1716) 14:1697–716. doi: 10.1080/21645515.2018.1458806 PMC6067889 29601253

[7] O'Shea MK Wilson D. Tuberculosis and the military. J R Army Med Corps. (2013) 159:190–9. doi: 10.1136/jramc-2013-000115 24109141

[8] Yuan Y Wang X Zhou Y Zhou C Li S. Prevalence and risk factors of latent tuberculosis infection among college students: a systematic review and meta-analysis. Public Health. (2022) 213:135–46. doi: 10.1016/j.puhe.2022.10.003 36410119

[9] Chen Q Yu S Rui J Guo Y Yang S Abudurusuli G. Transmissibility of tuberculosis among students and non-students: an occupational-specific mathematical modelling. Infect Dis Poverty. (2022) 11:117. doi: 10.1186/s40249-022-01046-z 36461098 PMC9716537

[10] Lu CW Lee YH Pan YH Chang HH Wu YC Sheng WH. Tuberculosis among migrant workers in Taiwan. Glob Health. (2019) 15:18. doi: 10.1186/s12992-019-0461-2 PMC6394038 30819237

[11] Charoensook P Upala P Anuwatnonthakate A Ruanjai T Apidechkul T. Pulmonary tuberculosis screening and quality of life among migrant workers northern Thailand. J Infect Dev Ctries. (2018) 12:1052–61. doi: 10.3855/jidc.10596 32027605

[12] Bennet R Nejat S Eriksson M. Effective tuberculosis contact investigation using interferon-gamma release assays. Pediatr Infect Dis J. (2019) 38:e76–8. doi: 10.1097/INF.0000000000002272 30882747

[13] Zhang HC Ruan QL Wu J Zhang S Yu SL Wang S. Serial T-SPOT.TB in household contacts of tuberculosis patients: a 6-year observational study in China. Int J Tuberc Lung Dis. (2019) 23:989–95. doi: 10.5588/ijtld.18.0252 31615605

[14] Fair E Miller CR Ottmani SE Fox GJ Hopewell PC. Tuberculosis contact investigation in low- and middle-income countries: standardized definitions and indicators. Int J Tuberc Lung Dis. (2015) 19:269–72. doi: 10.5588/ijtld.14.0512 25686131

[15] Zhao Y Chen M Xu C Zhang H. Technical guidelines for tuberculosis prevention and control in China In: Liu J, editor, vol. 2021. Beijing: People's Medical Publishing House (2021). 377.

[16] Shanghai Antituberculosis Association, Y.C.o.C.A.A. Editorial Board of Chinese Journal of Antituberculosis. Expert consensus on epidemiology investigation and scene disposal of tuberculosis in schools. Zhonghua Yu Fang Yi Xue Za Zhi. (2019) 41:247–51. doi: 10.3969/j.issn.1000-6621.2019.01.004 30841680

[17] Stata Corporation. Stata® 14 version 2. College Station: Texas: Stata Corporation (2015).

[18] Heymann D In: Heymann DL, editor. Control of communicable diseases manual. 19th ed. Washington: American Public Health Association (2011)

[19] Gao L Lu W Bai L Wang X Xu J Catanzaro A. Latent tuberculosis infection in rural China: baseline results of a population-based, multicentre, prospective cohort study. Lancet Infect Dis. (2015) 15:310–9. doi: 10.1016/S1473-3099(14)71085-0 25681063

[20] Velen K Shingde RV Ho J Fox GJ. The effectiveness of contact investigation among contacts of tuberculosis patients: a systematic review and meta-analysis. Eur Respir J. (2021) 58:2100266. doi: 10.1183/13993003.00266-2021 34016621

[21] Wu X Li Q Yang Y Zhang C Li J Zhang J. Latent tuberculosis infection amongst new recruits to the Chinese army: comparison of ELISPOT assay and tuberculin skin test. Clin Chim Acta. (2009) 405:110–3. doi: 10.1016/j.cca.2009.04.019 19410567

[22] Kaveh DA Garcia-Pelayo MC Hogarth PJ. Persistent BCG bacilli perpetuate CD4 T effector memory and optimal protection against tuberculosis. Vaccine. (2014) 32:6911–8. doi: 10.1016/j.vaccine.2014.10.041 25444816

[23] van der Meer JW Joosten LA Riksen N Netea MG. Trained immunity: a smart way to enhance innate immune defence. Mol Immunol. (2015) 68:40–4. doi: 10.1016/j.molimm.2015.06.019 26597205

[24] Kaufmann E Sanz J Dunn JL Khan N Mendonca LE Pacis A. BCG educates hematopoietic stem cells to generate protective innate immunity against tuberculosis. Cells. (2018) 172:176–190.e19 e119. doi: 10.1016/j.cell.2017.12.031 29328912

[25] Dye C. Making wider use of the world's most widely used vaccine: Bacille Calmette-Guerin revaccination reconsidered. J R Soc Interface. (2013) 10:20130365. doi: 10.1098/rsif.2013.0365 23904584 PMC3757998

[26] Liao Q Zheng Y Wang Y Ye L Liu X Jiao W. Effectiveness of Bacillus Calmette-Guérin vaccination against severe childhood tuberculosis in China: a case-based, multicenter retrospective study. Int J Infect Dis. (2022) 121:113–9. doi: 10.1016/j.ijid.2022.04.023 35429637

[27] Wang J Mi J Liang Y Wu X Zhang J Liu Y. RNA-seq analysis of the BCG vaccine in a humanized mouse model. Zoonoses. (2023) 3:1–10. doi: 10.15212/zoonoses-2022-0035

[28] Aspatwar A Gong W Wang S Wu X Parkkila S. Tuberculosis vaccine BCG: the magical effect of the old vaccine in the fight against the COVID-19 pandemic. Int Rev Immunol. (2022) 41:283–96. doi: 10.1080/08830185.2021.1922685 33960271 PMC8108189

[29] Gong W Wu X. Differential diagnosis of latent tuberculosis infection and active tuberculosis: a key to a successful tuberculosis control strategy. Front Microbiol. (2021) 12:745592. doi: 10.3389/fmicb.2021.745592 34745048 PMC8570039

[30] Cui X Gao L Cao B. Management of latent tuberculosis infection in China: exploring solutions suitable for high-burden countries. Int J Infect Dis. (2020) 92:S37–40. doi: 10.1016/j.ijid.2020.02.034 32114201

[31] Shah M Dorman SE. Latent tuberculosis infection. N Engl J Med. (2021) 385:2271–80. doi: 10.1056/NEJMcp2108501 34879450

